# The role of nitric oxide in memory is modulated by diurnal time

**DOI:** 10.3389/fnsys.2014.00059

**Published:** 2014-04-11

**Authors:** Stephanie L. Gage, Alan Nighorn

**Affiliations:** Department of Neuroscience, University of ArizonaTucson, AZ, USA

**Keywords:** moth, olfaction, antennal lobe, classical conditioning, proboscis extension

## Abstract

Nitric oxide (NO) is thought to play an important neuromodulatory role in the olfactory system. This modulation has been suggested to be particularly important for olfactory learning and memory in the antennal lobe (the primary olfactory network in invertebrates). We are using the hawkmoth, *Manduca sexta*, to further investigate the role of NO in olfactory memory. Recent findings suggest that NO affects short-term memory traces and that NO concentration fluctuates with the light cycle. This gives rise to the hypothesis that NO may be involved in the connection between memory and circadian rhythms. In this study, we explore the role of diurnal time and NO in memory by altering the time of day when associative-olfactory conditioning is performed. We find a strong effect of NO on short-term memory, and two surprising effects of diurnal time. We find that (1) at certain time points, NO affects longer traces of memory in addition to short-term memory; and (2) when conditioning is performed close to the light cycle switches—both from light to dark and dark to light—NO does not significantly affect memory at all. These findings suggest an intriguing functional role for NO in olfactory conditioning that is modulated as a function of diurnal time.

## Introduction

Nitric oxide (NO) is a free radical gas that can serve as both unconventional neurotransmitter and neuromodulator. Nitric oxide synthase (NOS) is highly expressed in the primary olfactory center in both vertebrates (olfactory bulb) and invertebrates (antennal lobe) (Bredt et al., 1991; Elphick et al., 1995; Muller and Hildebrandt, 1995; Hopkins et al., 1996; Kendrick et al., 1997; Nighorn et al., 1998; Fujie et al., 2002; Collmann et al., 2004). Given this widespread prominence, NO is thought to play a functional role in olfactory processing and behavior, yet the significance of this role is only beginning to be understood. We have previously shown that NO is necessary for short-term olfactory memory in the AL in the moth, *Manduca sexta* (Gage et al., 2013). These moths are nocturnal and heavily depend on their olfactory systems to find mates, feed, and find sites to lay eggs. During the nocturnal active period, NO levels are significantly higher in the antennal and optic lobes, suggesting that NO signaling is heightened at night and may play a phase-dependent role.

Circadian influence on memory and behavior is highly conserved (Gerstner et al., 2009). The repetitive nature of the light cycle that coincides with the availability of vital resources has led to a “timed” physiological environment (Gerstner, 2012). In this way, organisms experience physiological changes at the cellular and molecular level that are both circadian and seasonal, and ultimately lead to timed variations in behavior. These behavioral responses are often coordinated with regular and predictable stimuli present in the environment. For example, bees and moths forage at the time of day when pollen and sucrose are at peak levels (Baker, 1961; Guerenstein et al., 2004). Memory, which is intricately intertwined in behavior, has also evolved in a circadian context. Learning and memory are metabolically expensive, and it is widely believed that these mechanisms are conserved and function optimally when predictable resources are available (Dukas, 2008; Lyons, 2011; Gerstner, 2012). In essence, there is a “plasticity in plasticity”.

Though behavior and memory are controlled by the circadian clock, the nervous system can also modulate these effects (Gerstner, 2012). This variation is believed to exist to help animals adapt to a changing environment, such as the change in daylight hours throughout the year. This ability to adapt is suggested to be regulated by neuromodulators (Gerstner, 2012). Neuromodulators adjust sensory circuitry to account for changing conditions and are thought to optimize energy use in finding resources. NO could be an important neuromodulator in this process. NO is demonstrated to affect memory in many species and paradigms (Yamada et al., 1995; Muller, 1996; Kendrick et al., 1997; Prendergast et al., 1997a,b; Samama and Boehm, 1999; Yeh and Powers, 2005; Yabumoto et al., 2008; Kelley et al., 2010; Mutlu et al., 2011), and some reports also find direct effects of NO in the superchiasmatic nucleus (Ignarro, 2000) and in peripheral pacemakers (Bullmann and Stevenson, 2010).

The olfactory system provides an excellent opportunity to investigate the role of NO in memory. The primary olfactory center is organized similarly across phylogeny (Hildebrand and Shepherd, 1997), and NO is highly expressed in every primary olfactory center in which it has been examined (Bredt et al., 1991; Elphick et al., 1995; Muller and Hildebrandt, 1995; Hopkins et al., 1996; Kendrick et al., 1997; Nighorn et al., 1998; Fujie et al., 2002; Collmann et al., 2004). Olfactory learning and memory, especially in insects, is well-studied, and much is known about the behavior and molecular components (for reviews see Dukas, 2008; Davis, 2011; Giurfa and Sandoz, 2012). Olfactory memory also appears to be regulated by the circadian clock. Several reports reveal a circadian-dependent change in memory using olfactory conditioning. These effects have been demonstrated in the cockroach, *Leucophaea maderae* (Decker et al., 2007); in the soil dwelling nematode, *Caenorhabditis elegans* (Olmedo et al., 2012); and in the fruit fly, *D. melanogaster* (Lyons and Roman, 2008). It appears that the circadian clock regulates memory rather than olfactory responsiveness (Lyons and Roman, 2008; Lyons, 2011). Studies in rodents show that olfactory bulb neurons express functional and entrainable circadian rhythms that operate independently of the superchiasmatic nucleus (Granados-Fuentes et al., 2004). These rhythms in olfactory activity in both vertebrates and invertebrates appear to depend on *BMAL1* and *period* genes (Krishnan et al., 1999; Tanoue et al., 2004; Lyons and Roman, 2008; Granados-Fuentes et al., 2011; Hamada et al., 2011).

In this study, we utilize the olfactory system of *M. sexta* to study the role of NO in memory in relation to diurnal time. *M. sexta* demonstrate robust learning and memory in classical conditioning paradigms using the proboscis extension reflex (PER; Daly and Smith, 2000; Dacks et al., 2012; Gage et al., 2013). The olfactory behavior and ecology in the hawkmoth is well described and can be useful when interpreting olfactory memory with light/activity phase effects (Baker, 1961; Grant, 1983; Riffell et al., 2008). Although *M. sexta* is not a traditional model used in circadian rhythm biology, *period* expression is found in the photoreceptors in the compound eye, neurons in the optic lobe, and glial cells in the AL (Wise et al., 2002). We know that NOS is localized in the olfactory receptor neurons and sGC is expressed in all projection neurons, some local interneurons, and the serotonin immunoreactive neuron (Collmann et al., 2004). NO also exerts substantial effects at the physiological level in *M. sexta* that include: (1) a spatially focused increase in NO during odor stimulation (Collmann et al., 2004); (2) persistent basal levels in olfactory neurons that affect resting membrane conductance (Wilson et al., 2007); and (3) whole-cell current modulation (Higgins et al., 2012).

We ask two main questions in this study: (1) is there an optimal time of day for learning and memory in *M. sexta*; and (2) does the role of NO in memory change depending on the time of conditioning? To do so, we pair a microinjection surgery to manipulate NO levels in the AL with an appetitive, odor-associative conditioning paradigm. Conditioning is performed at different times around the clock that include 12 h of day, followed by 12 h of night. The PER is used to measure memory of the conditioned odor (CS). We tested olfactory memory at four time points after conditioning to account for both short-term, intermediate-term, and long-term memory traces. We present findings that suggest a role for NO in short-term and intermediate-term memory in *M. sexta* that is modulated by diurnal time.

## Materials and methods

### Animals

*M. sexta* (Lepidoptera: Sphingidae) were reared in the Department of Neuroscience at the University of Arizona. Animals were raised on an artificial diet and maintained under a long-day photoperiod regimen (17 h light:7 h dark) at 25°C at 50–60% relative humidity until early in pupal development. Most of pupal development occurs in constant darkness. Females at pupae stage 16 were transferred into a biological incubator (Model I-36 VL; Percival Scientific, Perry, IA, USA) and placed under a 12 h light:12 h dark cycle and kept at 25°C at 50–60% relative humidity. Five-day-old females were unfed after eclosion and used for all experiments. For all experiments, each animal was used only once.

### Pharmacology and Microinjection Surgery

NOS inhibitor, *N-*nitro-L-arginine methyl ester (L-NAME), was dissolved into physiological saline (150 mM NaCl, 3 mM KCl, 10 mM TES, pH 6.9) and used at a 15 mM concentration. L-NAME was chosen because it is the best characterized NOS inhibitor in this system. In *M. sexta*, this concentration was found to be the minimal effective dose in extracellular recording (Wilson et al., 2007) and also found to affect odor learning and memory (Gage et al., 2013).

Drug delivery into the ALs was accomplished via a microinjection surgery (Lei et al., 2009; Gage et al., 2013). Animals were restrained in a plastic tube, and an hourglass window was cut into the head capsule. The ALs were visualized by gently moving connective tissue with fine forceps. Quartz pipettes (o.d. 1.0 mm, i.d. 70 mm; Sutter Instruments, San Diego, CA, USA) were pulled with a Model P-2000 puller (Sutter Instruments) and clipped to allow solution passage. The pipettes were filled with either L-NAME or saline and manually injected into each AL (for visual see Gage et al., 2013) using a General Valve Picospritzer II (East Hanover, NJ, USA). The cut window was resealed with myristic acid (Sigma-Aldrich). The identity of the drug *versus* saline control was blind to both the experimenter performing the surgery and the experimenter observing behavior in all experiments.

In these experiments, NO levels were manipulated by inhibiting NOS rather than providing an exogenous source of NO. This is due to the unique glomerular anatomy of the olfactory system. In *M. sexta*, the glial cells surrounding each glomerulus provide a strong barrier to diffusion of NO making the bath application of a NO donor potentially problematic (Collmann et al., 2004; Higgins et al., 2012).

### Olfactory stimulation and appetitive conditioning

Hibiscus oil blend (diluted 1:1000 in mineral oil; Select Oils, Tulsa, OK, USA) was the odor used for appetitive conditioning. Hibiscus is not a reported host plant of hawkmoths and serves as a novel odor to gauge odor-associative learning and memory. Hibiscus was delivered by a solenoid-controlled air stream into an odor-containing glass syringe. Each syringe contained 10 µL of the odor on a piece of filter paper.

**Figure 1:**
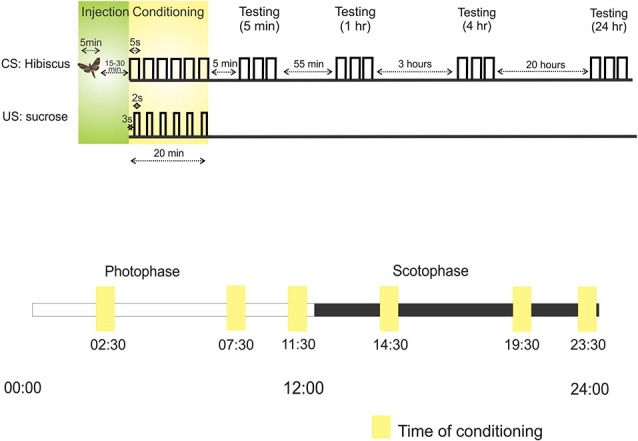
**Protocols outlining the sequence of injection, conditioning and testing**. Above: The first line, *CS: Hibiscus*, denotes the presentation of the conditioned stimulus (CS), hibiscus odor blend, through an air puff. Each raised step on this line refers to the presentation of hibiscus. For example, during conditioning, hibiscus was presented a total of six times to the antenna. The second line, US: sucrose, represents when sucrose, the unconditioned stimulus (US), was applied to the tip of proboscis relative to the CS odor puff. For example, sucrose was applied 3 s into the odor puff. Below: the protocol for testing learning and memory at different times over a 24 h period. Each square represents the time of conditioning in six separate experiments. Scotophase, represented by the black bar, denotes nighttime and photophase represented by the white bar, denotes daytime.

Appetitive conditioning was performed utilizing the PER. This is a feeding reflex that was originally discovered in honeybees (Takeda, 1961) that has also been used in *M. sexta* (Daly and Smith, 2000). Moths trained to associate an odor with a sucrose reward will extend their proboscis to the rewarded odor. This measure can be used in a number of paradigms and is especially useful to gauge odor learning and memory. In these experiments, moths were restrained in a plastic tube prior to surgery and conditioning. After surgery, a clear plastic tube was situated over the proboscis to secure a uniform position both to apply a sucrose reward (1 uL, 25% sucrose solution) and to observe maximum pumping motion and extension. Five-day-old moths were trained in a forward conditioning paradigm to associate hibiscus with the sucrose reward (Figure 1). The hibiscus-containing syringe was positioned approximately 5 cm from the antenna and delivered via a 5-s odor pulse. Three seconds into the pulse, sucrose was delivered to the tip of the proboscis using a pipette. This conditioning sequence was repeated six times, spaced 4 min apart. Multiple, spaced trials is a very robust form of conditioning that was employed to test shorter and longer forms of memory (Menzel, 2001).

### Learning and Memory

Proboscis extension to the conditioned odor was tested at four time points after conditioning: 5 min, 1 h, 4 h, and 24 h (Figure 1). These time points approximate memory traces that underlie short-term memory, short-term/intermediate-term memory, intermediate-term/long-term memory, and long-term memory, respectively (Davis, 2011).

### Diurnal time in learning and memory

We sought to test how an animal’s physiological time of day affects learning and memory and whether the role of NO in memory is affected. To do so, we chose six time points over a 24-h period divided into photophase (day; 00:00–12:00 h) and scotophase (night; 12:00–24:00 h) (Figure 1). Three time points in photophase were chosen: 02:30 h, 07:30 h, and 11:30 h; and three points in scotophase were chosen: 14:30 h, 19:30 h, 23:30 h.

These time points were chosen for three main reasons: (1) 14:30 was chosen because this time approximates the hours after dusk (2.5) when *M. sexta* are actively using their olfactory systems to find mates, feed, and find sites for oviposition (Gregory, 1963–1964; Yamamoto et al., 1969). 14:30 was the time point found in the Gage et al. (2013) study that showed a robust effect of NO in short-term memory. 02:30 was used as a photophase counterpoint to examine memory 2.5 h after photophase/sunrise; (2) 11:30 and 23:30 were chosen because each preceded the light cycle switch (from photophase to scotophase and from scotophase to photophase) by 30 min, potentially illuminating an association between memory and the impending light cycle change; and (3) 07:30 and 19:30 were chosen as mid-phase time points, both 7.5 h into photophase and scotophase. When animals were in scotophase, all manipulations including the surgical manipulations and olfactory conditioning were performed under dim red light so as not to affect the circadian clock. The potential effect of the surgery itself on the circadian clock was not tested.

### Statistical Analysis

All statistical analyses were performed using JMP 9.0.1 (SAS Institute, Cary, NC, USA). Proboscis extension reflexes were scored with a 1 or a 0 to employ parametric tests. A one-way ANOVA was performed with a *post-hoc* Tukey-Kramer HSD test. In all tests, α = 0.05, and a 95% confidence level was used. Data are expressed as means ± s.e.m.

## Results

### The role of NO in memory changes with the time of conditioning

**Figure 2:**
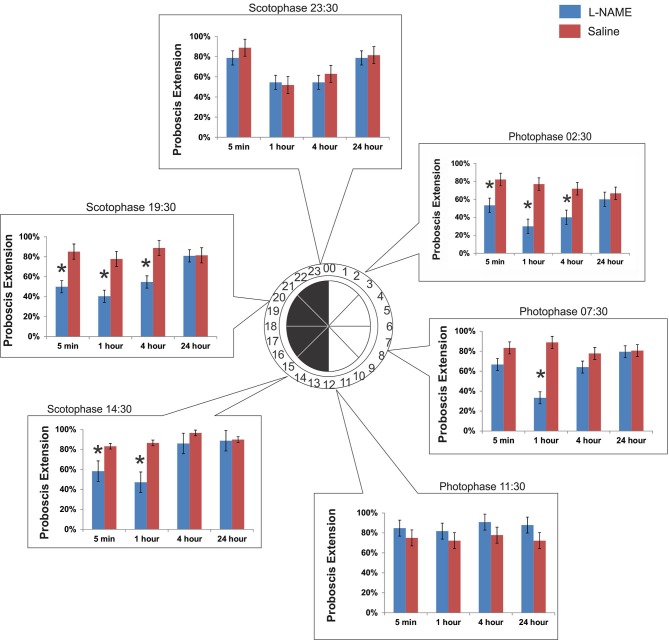
**The time of conditioning changes the effect of NO in memory**. Six separate experiments were performed to test the role of diurnal time in olfactory learning and memory and the role of NO. Six time points were chosen: 3 in photophase and 3 in scotophase. Conditioning began at the start of the time point (e.g., 14:30). After conditioning, animals were tested for their response to the CS (hibiscus odor) by observing the PER at 5 min, 1 h, 4 h, and 24 h. Asterisks denote significance between treatment groups (saline-injected and L-NAME injected) using a one-way ANOVA test. Total *N* = 136.

The time of olfactory conditioning influences the role of NO signaling in memory. Six conditioning times were chosen throughout the day governed by a 12 h light: 00:00–12:00/12 h dark: 12:00–24:00 cycle. At each conditioning time, animals were tested at 5 min, 1 h, 4 h, and 24 h after conditioning. What we found was a diunral, time-dependent change in the role of NO signaling in olfactory memory. Figure 2 encompasses all six time points discussed below:

#### Photophase 02:30

This time point was chosen to mimic the physiological time of day 2.5 h after sunrise in light conditions. Under light conditions, or photophase, *M. sexta* are at rest. When conditioned at 02:30, L-NAME-injected animals (L-NAME is a NOS inhibitor) show a significant decrease in proboscis extension when tested at 5 min (*F*_1,67_ = 7.09, *p* = 0.009, *N* = 23), 1 h (*F*_1,67_ = 18.92, *p* < 0.0001, *N* = 23), and 4 h (*F*_1,67_ = 7.61, *p* = 0.008, *N* = 23). There was no significant effect of L-NAME *versus* saline controls at 24 h after conditioning (*F*_1,67_ = 0.32, *p* = 0.57, *N* = 23).

#### Photophase 07:30

This time point was chosen to assess learning and memory mid-photophase, or 7.5 h after sunrise. At this time, *M. sexta* are at rest. When conditioned at 07:30, L-NAME-injected animals show a significant decrease in proboscis extension only at 1 h (*F*_1,73_ = 34.51, *p* < 0.0001, *N* = 25). Unlike at photophase 02:30, memory tested at 5 min (*F*_1,73_ = 2.78, *p* = 0.09, *N* = 25) and 4 h (*F*_1,73_ = 1.68, *p* = 0.19, *N* = 25) did not show significant differences with saline controls. There was no significant effect of L-NAME *versus* saline controls at 24 h (*F*_1,73_ = 0.01, *p* = 0.91, *N* = 25).

#### Photophase 11:30

This time point mimics the physiological time of day 30 min prior to dusk and the active evening period. When conditioned at 11:30, L-NAME-injected animals are not significantly different in proboscis extension than saline controls at any of the four time points tested (5 min: *F*_1,67_ = 1.02, *p* = 0.32, *N* = 23; 1 h: *F*_1,67_ = 0.88, *p* = 0.35, *N* = 23; 4 h: *F*_1,67_ = 2.22, *p* = 0.14, *N* = 23; 24 h: *F*_1,67_ = 2.63, *p* = 0.11, *N* = 23).

#### Scotophase 14:30

This time point mimics the physiological time of day 2.5 h after dusk. This time period is highly active. *M. sexta* can be found seeking mates, food, and sources to lay eggs. This time point was the time of conditioning in the Gage et al. (2013) study that reported the NO effects on short-term memory. When conditioned at 14:30, L-NAME injected animals show a significant decrease in proboscis extension at 5 min (*F*_1,64_ = 5.07, *p* = 0.028, *N* = 22) and 1 h post-conditioning (*F*_1,64_ = 13.09, *p* = 0.0006, *N* = 22). There was no significance found at 4 h (*F*_1,64_ = 2.21, *p* = 0.14, *N* = 22) or 24 h (*F*_1,64_ = 0.02, *p* = 0.89, *N* = 22).

#### Scotophase 19:30

This time point was chosen to assess learning and memory mid-scotophase. At this time, *M. sexta* are still active, but peak activity has begun to taper off (Gregory, 1963–1964). When conditioned at 19:30, L-NAME-injected animals show a significant decrease in proboscis extension with saline controls at 5 min (*F*_1,67_ = 9.80, *p* = 0.003, *N* = 23), 1 h (*F*_1,67_ = 10.36, *p* = 0.002, *N* = 23), and 4 h post-conditioning (*F*_1,67_ = 9.81, *p* = 0.002, *N* = 23). There was no effect of L-NAME *versus* saline controls at 24 h (*F*_1,67_ = 0.003, *p* = 0.96, *N* = 23).

#### Scotophase 23:30

This time point was chosen to assess learning and memory 30 min prior to sunrise. At this time, *M. sexta* are finding locations to hide and rest for the impending daytime hours. Similar to photophase 11:30 (just prior to the light switch to scotophase), L-NAME-injected animals conditioned at 23:30 do not show significant differences with saline controls at any time post-conditioning (5 min: *F*_1,58_ = 1.07, *p* = 0.30, *N* = 20; 1 h: *F*_1,58_ = 0.04, *p* = 0.84, *N* = 20; 4 h: *F*_1,58_ = 0.42, *p* = 0.52, *N* = 20; 24 h: *F*_1,58_ = 0.07, *p* = 0.79, *N* = 20).

### Individual memory windows are affected by diurnal time

**Figure 3:**
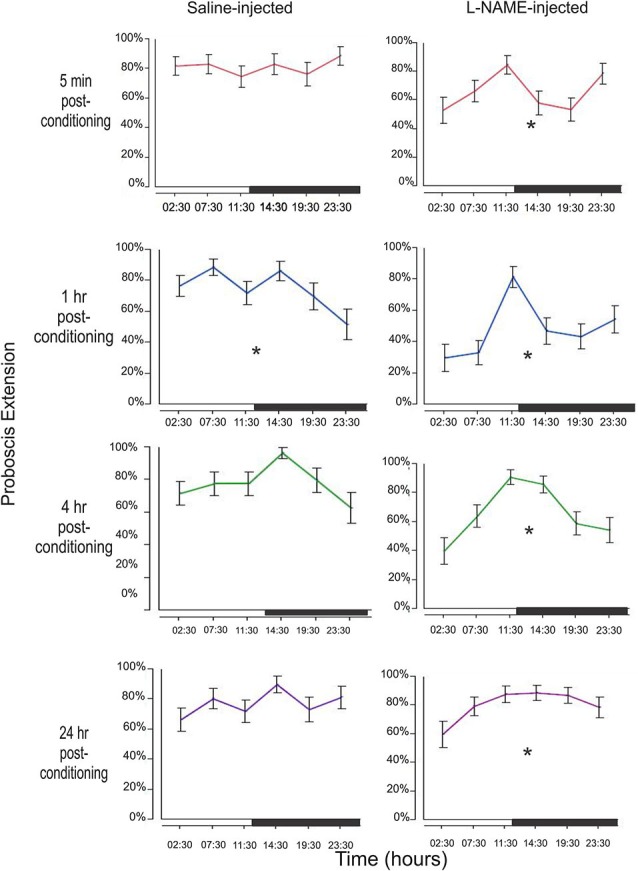
**Examination of memory by treatment and conditioning time throughout the day**. Memory was tested by observing the PER to the conditioned odor (CS, hibiscus) at 5 min, 1 h, 4 h, and 24 h after conditioning. This sequence was repeated at six time points throughout the day. Figure 3 looks at the effects of diurnal time on a single memory test (e.g., 5 min after conditioning). For example, the top left graph examines the 5 min results during each of the six time points tested around the clock. The black bar on each x-axis denotes the light cycle change (12 h light; 12 h dark) from photophase to scotophase beginning at 12:00. Asterisks denote a significant effect of conditioning time between at least one group/time point using a one-way ANOVA test. Total *N* = 136.

In addition to determining the treatment effect with L-NAME, we wanted to examine the effect of diurnal time both on the saline and L-NAME treated animals. Figure 3 examines each of the four time points (5 min, 1 h, 4 h, and 24 h) in both saline controls and L-NAME-injected animals to determine whether conditioning time is significant.

#### 5 min after conditioning

In this very short-term memory phase, there was no effect of diurnal time on saline injected animals (*F*_5,192_ = 0.51, *p* = 0.77). L-NAME treated moths, however, show a significant effect of conditioning time (*F*_5,204_ = 2.74, *p* = 0.02). L-NAME-injected moths appear to have a peak 5 min memory trace prior to both light cycle switches, with a trough in between time points (Figure 2).

#### 1 h after conditioning

This memory trace shows the most significant effect of diurnal time on the performance of control animals with a significant decrease just before the dark to light transition (*F*_5,192_ = 2.99, *p* = 0.01). L-NAME treated animals also exhibit a significant difference in proboscis extension (*F*_5,204_ = 5.08, *p* = 0.0002) with a profound change in response just before the light to dark transition.

#### 4 h after conditioning

The 4 h time point represents intermediate-term memory with molecular mechanisms that are likely to be different from either short-term or long-term memory (Bailey et al., 2008; Berry et al., 2008; Michel et al., 2012). At this interesting time point, saline-treated animals show a borderline statistical significance between time the 14:30 and 23:30 time points (*F*_5,192_ = 2.15, *p* = 0.06, Tukey-Kramer *post-hoc* shows significance *p* = 0.03). L-NAME-treated animals also showed a significant difference in proboscis extension among conditioning times (*F*_5,204_ = 6.12, *p* < 0.0001).

#### 24 h after conditioning

At 24 h after conditioning, widely viewed as the time frame for long-term memory formation (Davis, 2011), the saline-injected animals do not show changes in proboscis extension among conditioning times (*F*_5,192_ = 1.29, *p* = 0.27). L-NAME-injected moths, however, surprisingly show a small but statistically significant effect of conditioning time (*F*_5,204_ = 2.51, *p* = 0.03). This finding may suggest that NO plays a small role in long-term memory as well. Perhaps under a less robust conditioning paradigm, the effects of NO in long-term memory may be seen. In addition, it may also reveal a more global effect of diurnal regulation of long-term memory. Though no difference of long-term memory was observed in the control animals, perhaps the effect at 24 h in NOS-inhibited animals underscores a masked effect.

## Discussion

The diurnal time of conditioning modulates olfactory learning and memory in the moth, *M. sexta*. The effect of diurnal time is subtle in control animals, with the 1 h memory trace being affected by the time of conditioning only at the dark to light transition. In animals where NO signaling is inhibited, the effect of diurnal time in memory is more significant. We find that NO can affect multiple traces of memory, and that the importance of NO signaling is modulated by diurnal time. This modulation may be attributed to interactions with other neurotransmitters and neuromodulators, like serotonin, that should also be considered for circadian contributions to memory.

### NO affects longer memory windows in addition to short-term memory

We have previously shown that NO signaling is important for short-term memory in this olfactory conditioning paradigm (Gage et al., 2013). In this study we found that recall at the 4 h time window was affected by inhibition of NO signaling at two of the six time points tested: 19:30 and 02:30. The 4 h window reflects intermediate-term memory when compared to memory traces found in *D. melanogaster* (Davis, 2011). In *Aplysia*, this time window has been shown to be affected by both circadian time and NO in an operant conditioning paradigm (Michel et al., 2012, 2013). In that paradigm, inhibition of NOS interfered with conditioning but application of exogenous NO did not rescue the circadian-dependent inhibition. This suggests that changing NO levels could not explain the circadian-dependent effect on conditioning. The role of NO in *M. sexta* olfactory conditioning appears to be different. There is no evidence that changes in NO levels underlie the mild time-dependent effects on olfactory conditioning. Rather, the effect of NO itself on intermediate-term olfactory memory appears to be mediated by diurnal time. This reason for this effect on the role of NO is unclear, but fluctuating basal NO levels may play a role. We know that at 02:30, basal NO averages approximately 50 nM; and at peak activity time 12 h later at 14:30, basal NO is approximately 120 nM (Gage et al., 2013). These results may indicate that the effects of NO in memory formation are concentration-dependent. In moving forward, it would be helpful to determine basal NO levels at each conditioning time tested, and when 15 mM L-NAME is applied.

### NO does not affect memory at the light cycle switches

The second intriguing finding is that inhibition of NO signaling has no effect on olfactory memory at the light cycle switches. This finding was surprising given the robust role of NOS inhibition in short-term memory. At both light cycle switches, from light to dark (11:30) and from dark to light (23:30), NOS inhibition does not produce a significant change in memory compared with the saline controls (Figure 2). One interpretation may be that significant physiological changes are happening to prepare for the light cycle/phase shift. The role of NO in memory may be overshadowed by other forms of neuromodulation happening here. The 11:30 time point, which proceeds the nocturnal activity period, could be especially dominated by heightened physiological activity. This activity may be modulated by several neuromodulators. The ability to form and consolidate memories is also very important at this time. *M. sexta* are especially active 1–2 h after dusk, and perhaps this crucial time is too important to be regulated by a single neurotransmitter. An interesting possibility is that serotonin levels are high enough at these times that NO modulation is not necessary. Serotonin levels vary throughout the day (Kloppenburg et al., 1999) and the single serotonergic neuron in each AL expresses sGC making it a potential target of NO (Collmann et al., 2004). It is possible that one function of NO is to increase the level of serotonin in response to odor stimulation and this effect is not necessary if serotonin levels are already high.

## Conclusion

This study sought to shed light on two questions: (1) is there an optimal time of day for learning and memory in *M. sexta*; and (2) is the role of NO in memory modulated by the time of conditioning? In regards to the former, there does not seem to be a specific time of day in which learning and memory is optimal, but there is variation that appears phase-dependent. The role of NO in memory is also modulated by the time of conditioning. At some time points tested, NO affects longer traces of memory, in addition to short-term. There is also a curious lack of effect of NO in memory that appears specific to the light cycle switches. These unique roles of NO in memory may be the result of NO interaction with other neurotransmitters and modulators. Taken altogether, NO may be of special interest for studies examining the diurnal modulation of memory.

## Conflict of interest statement

The authors declare that the research was conducted in the absence of any commercial or financial relationships that could be construed as a potential conflict of interest.
